# Destruction of halogen-containing pesticides by means of detonation combustion

**DOI:** 10.1007/s11356-012-1277-2

**Published:** 2012-11-06

**Authors:** Jolanta Biegańska

**Affiliations:** Faculty of Energy and Environmental Engineering, Department of Technology and Installations for Waste Management, Silesian University of Technology, 18, Konarskiego St, 44-100 Gliwice, Poland

**Keywords:** Pesticides, Detonative decomposition, Waste disposal

## Abstract

Pesticides that contain a halogen functional group have been destructed by means of detonative combustion. The following compounds were examined: (1) atrazine—2-chloro-4-ethylamino-6-isopropylamino-1,3,5-triazine—herbicide; (2) bromophos—*O*,4-bromo-2,5-dichlorophenyl *O*,*O*-dimethyl phosphorothioate—insecticide; (3) chloridazon—5-amino-4-chloro-2-phenylopyridazin-3(2H)-one—herbicide; (4) linuron—3-(3,4-dichlorophenyl)-1-metoxy-1-methylurea—herbicide; (5) metoxychlor—1,1,1-trichloro-2,2-bis(4-metoxyphenyl)ethane—insecticide and acaricide; and (6) trichlorfon—dimethyl 2,2,2-trichloro-1-hydroxyethylphosphonate—insecticide. Explosive material has been produced on the basis of ammonium nitrate, which served as an oxidizer while the pesticides were used as fuels. Composition of the explosive was adjusted in such a way as to respect thermodynamic parameters. Detonative decomposition of the mixtures has been carried out in shot-holes pre-drilled in soil. Efficiency of the pesticide decomposition has been examined with gas chromatography in order to determine pesticides residues in the environment. It was found that for some, the amount of pesticides in some compounds in the analyzed samples after decomposition was below the determination threshold of the applied method.

## Introduction

Organochlorine pesticides are dispersed in the environment—they even appear in locations that are far away from agricultural and industrial areas. Number of organochlorine compounds that have been identified in the environment (Skrbic and Durisic-Mladenovic 2007; Hosie 2000; Pavoni et al. 2006; Grynkiewicz et al. 2003) amounts to about 15,000, 50 % of which are difficult to decompose. Organochlorine compounds are accumulated in animal fat tissue.

Biodegradation of organochlorine is a complex process and active chemical reactions may lead to formation of metabolites that are even more toxic than the original reactants. Furthermore, pesticide wastes that have been stored in burial grounds (Wołkowicz et al. 2005; Gałuszka et al. 2011) pose the most critical threat to the environment. Organochlorine compounds (Gałuszka et al. 2011) constitute about 30 % of all the pesticides that are stored in the burial grounds in Poland.

The literature sources describe various methods of the waste pesticides disposal (Salvestrini et al. 2002; Seiber 1992; Mahamuni and Adewuyi 2010; Legrini et al. 1993; Kim et al. 2011). Biological, physical, chemical, and thermal methods are depicted. Problem of pesticide wastes is the never-ending subject of research, as new methods and ways of neutralization and detoxication of hazardous deposits are still searched for. The present paper describes decomposition of several halogen-containing pesticides by means of detonative combustion.

## Experimental part

It was assumed that ammonium-nitrate fuel oil (ANFO) would be used as a heterogeneous explosive material, where porous ammonium nitrate would act as oxidizer and the pesticide would be used as fuel. Containers were filled with explosive mix composed of *a*% of NH_4_NO_3_ and *b*% flammable component (pesticide-fuel).

Composition of mixtures that contain some porous ammonium nitrate and the examined pesticide have been designed in such a way that the obtained explosive reach density of *d* = 0.900 kg/dm^3^ and oxygen balance *B = 0* %. For the designed explosive mixtures, the thermodynamic parameters have been calculated in order to determine temperature of the explosions, considering that the temperature should be high enough to assure decomposition of the pesticide.

The obtained results are presented in Table 1. For comparison, the table shows parameters of the typical explosive ANFO produced from ammonium nitrate and paraffin oil.

**Table 1:** Thermodynamic parameters of explosives produced on the basis of ammonium nitrate and pesticide-containing materials

Sample no.	ANFO	1	2	3	4	5	6
Name of the flammable component	Paraffin oil	Atrazine	Bromophos	Chloridazon	Linuron	Metoxychlor	Trichlorfon
Composition of the explosive							
Flammable component [%]	5.47	10.49	17.54	10.79	12.91	10.33	23.45
NH_4_NO_3_ [%]	94.53	89.51	82.46	89.25	87.09	89.67	76.55
Rated number of moles of individual elements per 1 kg of the explosive							
Carbon	3.88	3.90	3.83	4.85	4.66	4.78	3.64
Hydrogen	55.36	51.54	45.04	48.48	48.71	49.29	45.55
Oxygen	35.45	33.55	32.34	33.94	33.68	34.21	32.33
Nitrogen	23.63	58.35	20.60	23.76	22.80	22.41	19.13
Others		0.49 Cl_2_	0.48 S	0.49 Cl_2_	1.04 Cl_2_	0.89 Cl_2_	0.91 P
			0.48 P				2.77 Cl_2_
			0.96 Cl_2_				
			0.48 Br_2_				
Chemical composition of the explosion products [mole/kg of the explosive]							
Carbon dioxide	3.88	3.90	3.83	4.85	4.66	4.78	3.64
Water (gaseous)	27.68	25.77	22.52	24.24	24.36	24.65	22.77
Nitrogen	11.82	29.17	10.30	11.88	11.40	11.20	9.56
Others		0.24 P_2_O_5_	0.48 Cl_2_	0.24 Cl_2_	0.52 Cl_2_	0.45 Cl_2_	0.46 P
			0.24 Br_2_				1.37 Cl_2_
			0.48 Cl2				
			0.24 Br2				
Specific volume of the explosion products, *V*_*o*_ [dm^3^/kg]	972.16	1,323.35	848.35	923.56	917.40	920.58	836.57
Heat of combination for the explosive, *Q*_*o*_ [kJ/kg]	4,444.64	4,620.93	4,558.55	4,563.37	4,561.83	6,142.92	4,203.07
Total heat of combination for the explosion products, *Q*_*p*_ [kJ/kg]	8,194.82	7,737.69	7,454.04	7,747.16	7,701.37	7,818.15	7,624.24
Heat of explosion *Q*_*w*_ [kJ/kg]	3,750.17	3,116.76	2,895.50	3,183.80	3,139.53	1,675.23	3,421.17
Concentration of energy, *E*_*v*_ [kJ/dm^3^]	3,375.16	2,805.08	2,605.96	2,865.42	2,825.58	1,507.71	3,079.05
Temperature of explosion, *T*_*w*_ [K]	2,753	2,026	2,459	2,462	2,558	1,597	2,775
Average specific heat of gaseous products of explosion, *c*_*v*_ [J/mol K]	34.85	30.09	33.56	35.29	33.56	30.79	33.86
Exponent of the adiabatic curve, k	1.24	1.28	1.25	1.20	1.25	1.27	1.25
Explosion pressure, *P*_*w*_ [MPa]	893.76	895.61	696.54	759.21	783.55	490.88	775.48
Ideal work of explosion, *A* [kJ/kg]	3,100.00	2,689.76	2,403.26	2,464.26	2,618.37	1,400.50	2,849.83
Specific energy, *f* [kJ/kg]	993.06	995.12	773.93	843.57	870.62	545.43	861.65

### Detonative decomposition in soil

Plastic PET container of 180-mm diameter and 5-dm^3^ capacity was used to decompose pesticides by means of detonative combustion. The explosive material was introduced to the container. A 2-kg load of explosive (ANFO) and pesticide was prepapred. Then the load was furnished with the NONEL detonator and placed into a previously prepared shot hole in the soil at the depth of 120 cm, protected by stemming.

Next, a blasting cap tied to a blasting fuse was attached to NONEL cord. Such a load, placed in the hole, was fired. The procedure—calculations, blast-hole dimensioning, soil sampling—was similar to the procedure described in Biegańska (2005) for the decomposition of DNOC in soil.

After detonation of the explosive, soil samples were taken for analysis. For sampling, five boreholes were made with a manual drill (gimlet). Each borehole had a diameter of 10 cm and the depth of 140 cm (with reference to the level before detonation).

The soil, sampled from each borehole, was placed on plastic foil and thoroughly mixed. Such samples were taken for analysis.

### Preparation of samples for determination of pesticide content

Weighted samples of soil (50 g) were drenched with two portions of chloromethane of 50 cm^3^ each, one after another. Afterwards, the extraction in an ultrasonic bath was carried out (Ashley 1995). The extracts were filtered and dried in a vacuum evaporator. The dry residues were standardized by dissolving in 10 cm^3^ of acetone. After such preparation, the sample was analyzed for the content of biologically active substances.

### Estimation of pesticide content in soil by means of gas chromatography method

The amount of pesticides remaining in extracts prepared from the soil samples was determined using gas chromatography method. A gas chromatography analysis has been performed by Varian type 3400, equipped with the electron capture detector and an integrator, marked with the IBDH symbol for automatic data processing. Chromatographic separation of components was carried out in a capillary column made of silica (silicon dioxide) with chemical connection to the liquid phase. The capillary column was factory labeled as DB-5. Internal diameter of the column was 0.25 mm and thickness of the phase film was 0.25 μm. The column was 30 m long. Thermal conditions of the separation were as follows: temperature of the feeder was 553 K and temperature of the detector was 573 K. Over the first 5 min temperature of the column was set at 353 K, then programmed to gradual heating at the rate of 10 grads/min, up to 563 K. Helium, at its flow rate of 1 ml/min, served as the carrier gas. The component to be determined was identified by comparing retention time of given substance.

The extracts prepared from the soil contain remaining amounts of pesticides, if any, but also other organic compounds, which become volatile under thermal conditions of gas chromatography. That is why the chromatograms present peak areas that correspond respectively to organic components of soil and the investigated pesticides. Approximately half of the compounds to be determined elute outside the elution range for the soil components. The other half elutes during the same or similar retention time.

In the first case, the detector emits signal in the form of a peak and numerical value which represents the amount of the determined pesticides. Lack of that signal should be the understood as the proof that no pesticide was detected.

In the second case, signal from the detector that corresponds to the compound to be determined may be located among signals which correspond to particular components of soil. Such signal when identified is used directly to mark the amount. The retention times may be so similar that they merge into a common signal. In such case, it is required to calculate values which correspond to the component to be determined from the overlaping part.

The above calculations are founded on methods used in gas chromatography to mark hidden components. They are based on the proportion of peak amplitudes or numerical signals selected separately for each case. These peaks (values) should be identified on chromatograms from a reference test sample and the analyzed one. The blind sample is an extract from soil that is used for technological experiments, whereas the analyzed sample is an extract of the same soil mixed with the pesticide and decomposed with the explosive.

Serial studies on chromatography show that the chromatogram for the analyzed sample is considered to be a “blind” chromatogram, but without the section that covers the peak of the component to be determined and with the peaks directly preceding and following the analyzed peak. As the components to be determined elute at different retention times, such an approach is reasonable and commonly used.

Signal value for the component to be determined was calculated from the common value for two or more components on the basis of the proportion rates in the following way: a peak area—point of reference was selected on the chromatogram for the reference sample. It had the retention time slightly different than the retention time of the component to be determined. The numerical value of the signal equaled *a*. Similarly, a peak or a group of peaks were selected that could include the peak area of the components to be determined with the numerical value of signals equal to *b*. The ratio *a/b* has a specific value. The same peaks on the chromatogram of the analyzed sample reach the respective numerical values of *c* and *d*. The ratio *a/b = c/x* gives the value of *x* if the tested sample does not contain any amounts of the component to be determined. The difference *d* − *x = g* is value of the hidden signal of the component to be determined.

Following the procedure, certain sample amount of solution was introduced into the device in order to create chromatogram. Then, the chromatogram was drawn and processed according to the description. The numerical values of the signals either read from the detector or calculated for the components to be determined were introduced as input data for the integrator that stored in its memory relevant parameters of calibration curves. Finally, results of determination of the components were given through mathematical computations.

## Results and discussion

**Fig. 1:**
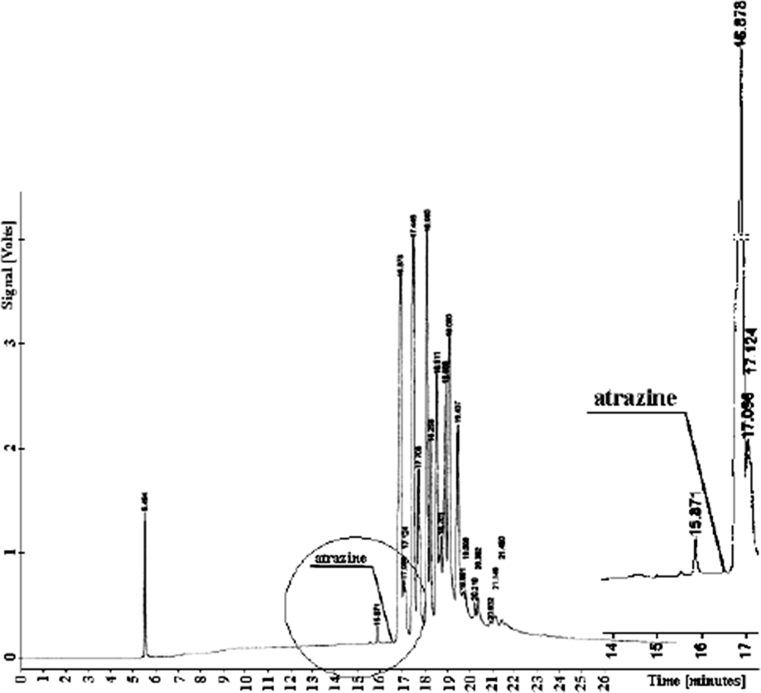
Chromatogram of atrazine

The atrazine elutes from the column at *t*_*R*_ *= 16.576*, earlier than the soil component. Therefore, the analytic procedure is simple—Fig. 1 shows chromatograms from the determination of atrazine in samples of neutral and acid extraction. The chromatograms printouts showed neither peak areas nor detector signals that would reflect pesticide residues.

**Fig. 2:**
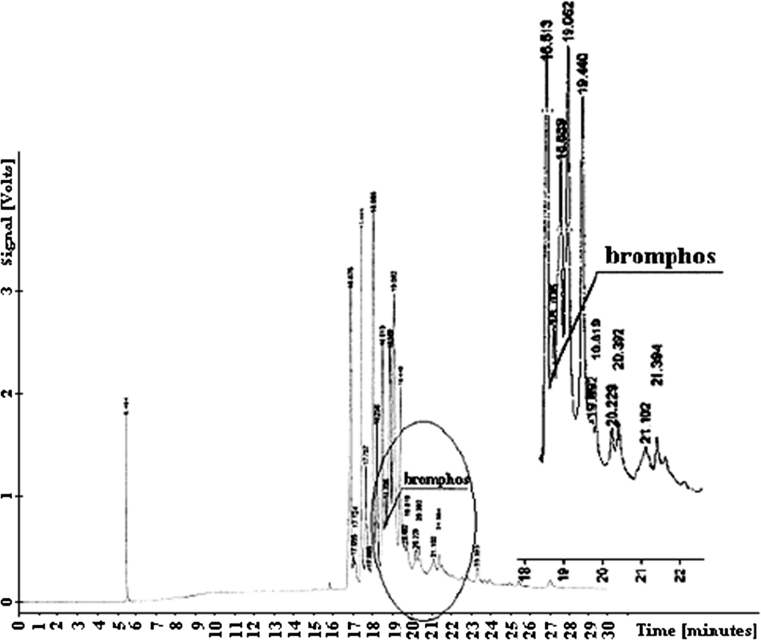
Chromatogram of bromophos

Bromophos elutes from the column at *t*_*R*_ *= 18.678* together with the group of soil components. Hidden signal was calculated from the ratio of the reference peak at *t*_*R*_ *= 18.065* and the sum of peak values at *t*_*R*_ *= 18.513*, *t*_*R*_ *= 18.706* and *t*_*R*_ *= 18.889*, considering chromatograms for the blind sample and analyzed samples. Figure 2 presents chromatogram of determination of that compound.

**Fig. 3:**
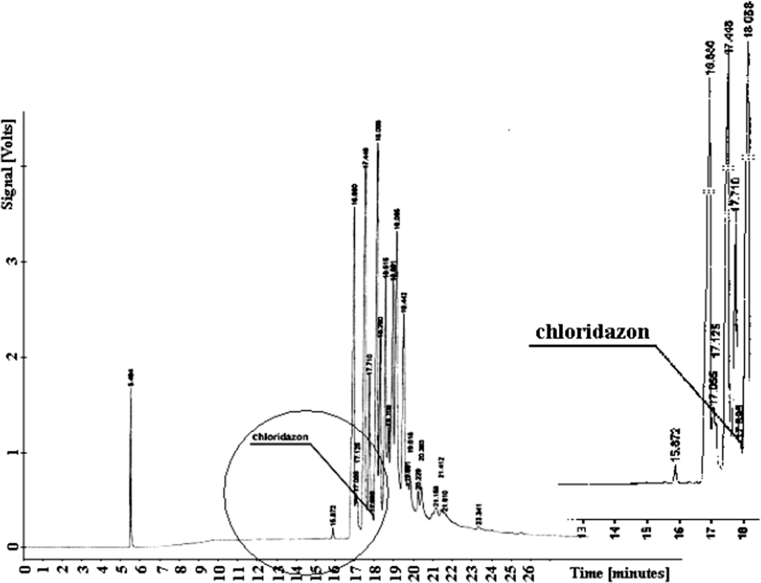
Chromatogram of chloridazon

**Fig. 4:**
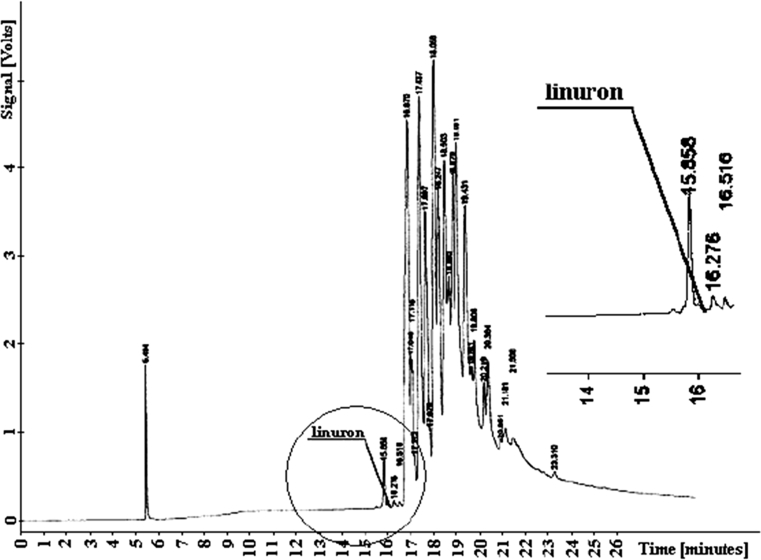
Chromatogram of linuron

Chloridazon elutes from the column at *t*_*R*_ *= 17.895* together with the group of soil components (Fig. 3). Linuron elutes from the column earlier than the coil components at *t*_*R*_ = 16.072. The early elution is a factor that facilitates analysis of the samples. The chromatograms for determining content of that compound are shown in Fig. 4. The diagrams present no peaks that would refer to linuron and no respective detector signal printouts could be obtained.

**Fig. 5:**
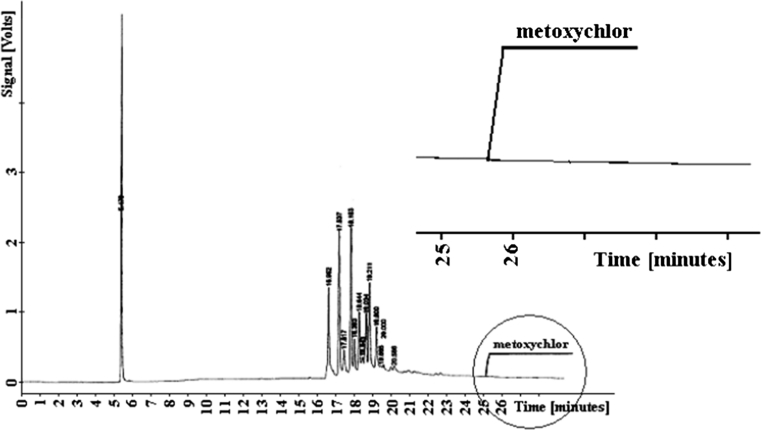
Chromatogram of metoxychlor

Metoxychlor elutes with the retention time *t*_*R*_ *= 25.572* after the soil components. The related chromatogram for the component remainder with the retention parameter marked on the diagram is shown in Fig. 5.

**Fig. 6:**
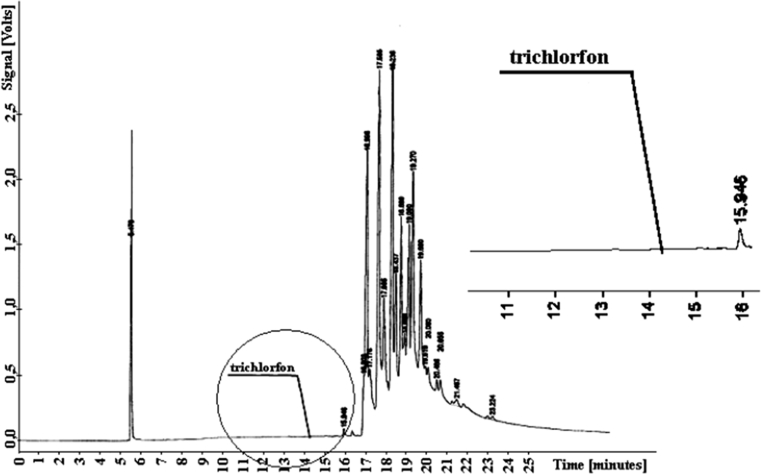
Chromatogram of trichlorfon

The trichlorfon elutes from the column at *t*_*R*_ *= 14.244*, earlier than the soil components. Hence, determination of that component presents no difficulty. Figure 6 shows a typical chromatogram with the retention parameter of the compound marked.

Results of determining the residual content of crop protection agents in soil have been summarized in Table 2. The chromatography showed that residues of only two biologically active components of pesticides, namely bromophos and chloridazon were present in soil after detonative combustion.

**Table 2:** Residual content of crop protection agents in the soil after their detonative decomposition

Sample no.	Preparation (commercial name)	Active agent	Result, mg/kg	Remarks
1	Gesaprim 50 WP	Atrazine	pgo	dt = 7.5 mg/kg
2	Nexion EC 40	Bromophos	3.64	dt = 1.0 mg/kg
3	Pyramin 65 WP	Chloridazon	9.28	dt = 4.0 mg/kg
4	Afalon 50 WP	Linuron	bdt	dt = 2.75 mg/kg
5	Liquid Metox 30	Methoxychlor	bdt	dt = 5.0 mg/kg
6	Liquid Foschlor 25	Trichlorfon	bdt	dt = 0.5 mg/kg

*dt* determination threshold for the biologically active agent, *bdt* below the determination threshold, *pgo* = adt = above the determination threshold

For the majority of the pesticides, detonative decomposition left insignificant amounts of biologically active compounds. Amounts of chemicals in samples identified after the detonations were below the determination threshold. This proves that the hazardous substances have been decomposed to a very high degree.

The achieved results allow to draw a conclusion that the waste pesticides that contain halogens can be used to manufacture explosives of ANFO type widely used in open-cut mining industry for getting limestone.
